# Role of C-Reactive Protein as a Predictor of Early Revascularization and Mortality in Advanced Peripheral Arterial Disease

**DOI:** 10.3390/jcm14030815

**Published:** 2025-01-26

**Authors:** Giuseppe Di Stolfo, Mario Mastroianno, Michele Antonio Pacilli, Giovanni De Luca, Carlo Rosario Coli, Ester Maria Lucia Bevere, Gabriella Pacilli, Domenico Rosario Potenza, Sandra Mastroianno

**Affiliations:** 1 Cardiovascular Department, Fondazione IRCCS Casa Sollievo della Sofferenza, 71013 San Giovanni Rotondo, Italy; ma.pacilli@operapadrepio.it (M.A.P.); g.deluca@operapadrepio.it (G.D.L.); c.coli@operapadrepio.it (C.R.C.); esterb94@hotmail.it (E.M.L.B.); d.potenza@operapadrepio.it (D.R.P.); s.mastroianno@operapadrepio.it (S.M.); 2 Scientific Direction, Fondazione IRCCS Casa Sollievodella Sofferenza, 71013 San Giovanni Rotondo, Italy; m.mastroianno@operapadrepio.it; 3 Emergency Department, Fondazione IRCCS Casa Sollievo della Sofferenza, 71013 San Giovanni Rotondo, Italy; gabriella.pacilli@operapadrepio.it

**Keywords:** C-reactive protein, peripheral revascularization, peripheral arterial disease, major adverse cardiovascular events, major adverse peripheral events

## Abstract

**Background:** Elevated high-sensitivity C-reactive protein (hsCRP) levels are associated with poor cardiovascular outcomes, particularly in patients with advanced peripheral arterial disease (PAD). This study aimed to assess the impact of hsCRP on clinical characteristics and long-term outcomes in a cohort of PAD patients. **Methods:** A total of 346 patients with advanced PAD were enrolled and stratified into two groups based on their median hsCRP level (Group 1: <0.32 mg/dL, Group 2: >0.32 mg/dL). The patients were followed for a mean of 102.70 ± 44.13 months. Their clinical characteristics, comorbidities, and long-term cardiovascular events, including myocardial and/or peripheral revascularization, ischemia, and death, were analyzed. This study evaluated two composite endpoints: major adverse cardiovascular events (MACEs) and major adverse peripheral events (MAPEs). MACEs comprised fatal cardiovascular events, cerebral ischemia, cardiac infarction, myocardial revascularization, acute peripheral arterial occlusion, and peripheral reperfusion. MAPEs included carotid reperfusion, acute peripheral arterial occlusion, and lower limb revascularization. **Results:** The patients in Group 2 had a higher body mass index, waist circumference, and waist–hip ratio compared to those in Group 1 (all *p* < 0.05). Inflammatory markers, including fibrinogen and the erythrocyte sedimentation rate, were significantly elevated in Group 2 (both *p* < 0.01). While the overall incidence of peripheral revascularization was similar between groups, these interventions occurred significantly earlier in Group 2 (28.24 ± 38.87 months vs. 67.04 ± 49.97 months, *p* = 0.004; HR: 2.015, 95% CI: 1.134–3.580, *p* = 0.017). The MAPEs were comparable in number, but occurred earlier in Group 2 (36.60 ± 37.35 months vs. 66.19 ± 48.18 months, *p* < 0.01; HR: 1.99, 95% CI: 1.238–3.181, *p* = 0.004). Similarly, the MACEs had an earlier onset in Group 2 (40.31 ± 38.95 months vs. 55.89 ± 46.33 months, *p* = 0.04; HR: 1.62, 95% CI: 0.983–1.987, *p* = 0.062). A total of 169 deaths were recorded during the follow-up. Group 2 exhibited a significantly higher mortality rate (56% vs. 42%, *p* < 0.01) and an earlier trend in mortality (76.58 ± 43.53 months vs. 84.86 ± 5.18 months), although this difference did not reach statistical significance (*p* = 0.22). **Conclusions:** Elevated hsCRP levels (>0.32 mg/dL) are associated with a worse clinical profile and earlier adverse events in patients with advanced PAD. Group 2 experienced significantly earlier peripheral revascularization, MACEs, and MAPEs. The mortality rates were also significantly higher, highlighting the prognostic value of hsCRP in this population.

## 1. Introduction

High-sensitivity C-reactive protein (hsCRP) is a well-established biomarker of systemic inflammation, and its elevation has been consistently linked to an increased risk of cardiovascular events, including myocardial infarction and stroke [1,2]. hsCRP is synthesized by the liver in response to inflammatory cytokines, particularly interleukin 6 (IL-6), and plays a pivotal role in both acute and chronic inflammatory responses [3,4]. In cardiovascular disease, high hsCRP levels reflect underlying inflammation that may exacerbate atherosclerosis, endothelial dysfunction, and plaque instability, leading to adverse clinical outcomes [5,6].

Peripheral arterial disease (PAD) is a manifestation of systemic atherosclerosis that primarily affects the lower extremities and is characterized by the progressive narrowing and obstruction of peripheral arteries [7,8]. Patients with advanced PAD are at a significantly increased risk of cardiovascular events due to the shared pathophysiological mechanisms of inflammation and atherosclerosis [9]. Inflammation, as reflected by elevated CRP, has been shown to contribute to the progression of PAD, worsening limb ischemia and increasing the risk of cardiovascular morbidity and mortality [10].

Several studies have emphasized the prognostic value of hsCRP in predicting cardiovascular outcomes across various patient populations [5,11,12]. However, its specific role in patients with advanced PAD remains underexplored, particularly in relation to limb-specific outcomes, such as lower limb revascularization, ischemia, the amputation of a lower limb, and carotid revascularization treatment. Given the dual threat posed by cardiovascular and limb-related complications in PAD, understanding how hsCRP levels correlate with these outcomes is crucial for improving risk stratification and therapeutic decision making [13].

In this study, we assessed the hsCRP levels in a cohort of 346 patients with advanced PAD to explore their association with long-term cardiovascular and peripheral artery-related outcomes. Specifically, we analyzed the incidence of major adverse cardiac events (MACEs), peripheral revascularizations, and major adverse peripheral events (MAPEs), defined as carotid/lower limb revascularization or amputation, over a mean follow-up period of 102.70 ± 44.13 months. Additionally, we examined the timing of these events and the overall mortality in relation to the baseline hsCRP levels.

## 2. Materials and Methods

We conducted a longitudinal cohort study of cardiovascular patients. The research protocol (TOMM40, version 11, 13 January) was approved by the ethics committee of “Casa SollievodellaSofferenza” Hospital and conducted in compliance with the Declaration of Helsinki, good clinical practice guidelines, and the STROBE (Strengthening the Reporting of Observational Studies in Epidemiology) standards.

This study comprised 346 elderly patients with advanced atherosclerosis (272 males and 74 females; mean age, 72.11 ± 7.90 years) who consecutively attended our clinic from 1 November 2011 to 31 October 2023. The study subjects were recruited with written informed consent. The inclusion criteria encompassed individuals of Caucasian ethnicity with severe atherosclerotic disease, characterized by carotid stenosis > 50% on an ultrasound Doppler assessment and/or peripheral arterial disease classified as Fontaine stage II (claudication) or stage III (ischemic rest pain). The exclusion protocol eliminated cases with mild carotid atherosclerosis (<50% stenosis), subclinical arterial disease (Fontaine stage I), critical limb ischemia (Fontaine stage IV), or an active malignancy with projected survival under 6 months.

Clinical data acquisition included pharmacological interventions, neoplastic conditions, nicotine use, obesity, lipid disorders, hypertensive disease, and diabetes mellitus type 2, utilizing the WHO and ATP III diagnostic guidelines [14]. Baseline cardiovascular events were documented from clinical records, encompassing cerebrovascular incidents, acute coronary syndromes, peripheral arterial interventions (carotid and lower extremity), and coronary revascularization procedures prior to the study’s initiation.

At the time of enrollment in the clinical study, the patients underwent laboratory examinations for blood chemistry and a urine analysis, including measurements of high-sensitivity C-reactive protein (hsCRP), fibrinogen, cholesterol, triglycerides, glucose, creatinine, vitamin D, potassium, and albumin. The urine testing assessed the creatinine and microalbumin levels, with the albumin-to-creatinine ratio (ACR) calculated from these values. All the hematochemical and urinary analyses were conducted in our institute’s clinical laboratory. Insulin sensitivity was evaluated using two homeostatic model assessment indices: HOMA-IR for insulin resistance and HOMA-β for beta-cell function. Kidney function was assessed by calculating the estimated glomerular filtration rate (eGFR) using the modification of diet in renal disease (MDRD) formula [15].

Vascular elasticity was evaluated using non-invasive measurements with the AngE System (Sonotechnik, Austria) to determine the pulse wave velocity (PWV) and augmentation index. For accuracy, the PWV and augmentation index measurements were taken twice and averaged for the final analysis. Cardiac imaging was performed using the MyLab50 Ultrasound System (Esaote). During echocardiography, the patients were positioned in left lateral decubitus while the following parameters were measured in the parasternal long-axis view: the interventricular septal thickness, left ventricular posterior wall thickness, and end-diastolic left ventricular diameter. The left ventricular mass was computed using the Devereux formula and normalized to body surface area to obtain the ventricular mass index. The cardiac electrical activity was assessed using standard 12-lead electrocardiography, measuring key intervals and conduction parameters, including the PR interval, QRS duration, QTc interval, and presence of a bundle branch block.

The patients were followed for an average of 102.70 ± 44.13 months. During this period, deaths, cerebrovascular accidents, myocardial infarctions, carotid revascularizations, acute lower limb ischemia, lower limb procedures of revascularization, and myocardial revascularizations were recorded. We also evaluated the following composite endpoints: peripheral revascularization, which included carotid and/or lower limb procedures; major adverse cardiovascular events (MACEs), which included fatal cardiovascular events, cerebral ischemia, cardiac infarction, myocardial revascularization, acute peripheral arterial occlusion, and peripheral reperfusion; and major adverse peripheral events (MAPEs), which included carotid or lower limb revascularization treatment and lower limb amputations. We grouped the causes of death into three groups: cardiovascular events (occurring due to acute cerebral ischemia, a fatal myocardial infarction, sudden death, or heart failure), cancer, and other causes (e.g., sepsis, trauma, severe anemia, acute respiratory failure, or severe renal failure).

Based on the median hsCRP value of 0.32 mg/dL, the participants were divided into two groups: those with low hsCRP (Group 1, hsCRP < 0.32 mg/dL), showing mean levels of 0.30 ± 0.03 mg/dL, and those with a higher hsCRP (Group 2, hsCRP ≥ 0.32 mg/dL), with mean levels of 0.90 ± 1.10 mg/dL.

### Statistical Analysis

The statistical analysis was conducted using the R software 4.3. For descriptive statistics, continuous variables were expressed as the mean ± standard deviation, while categorical variables were presented as absolute frequencies and percentages. Between-group comparisons for categorical variables were performed using Pearson’s χ^2^ test. For numerical data, normality was first verified using the Kolmogorov–Smirnov test, followed by between-group comparisons using unpaired two-tailed *t*-tests. Changes in medical treatment from pre-enrollment to study initiation were assessed using paired Student’s *t*-tests. The statistical significance was set at *p* < 0.05. The cardiovascular event risk was evaluated using Cox proportional hazards regression, with the results expressed as hazard ratios (HRs) and their corresponding 95% confidence intervals (95% CIs). The HRs were computed both unadjusted and adjusted for common risk factors, including BMI, waist–hip ratio, fibrinogen, ESR, HOMA-IR, and HOMAβ. Kaplan–Meier survival curves were employed to illustrate the rate of being event-free during follow-up.

## 3. Results

### 3.1. Baseline Characteristics of the Overall Population

A total of 346 patients with advanced peripheral arterial disease (PAD) were enrolled in the study. The majority of the participants were male (79%), with a mean age of 72.11 ± 7.90 years. Among these patients, 84% exhibited hemodynamically significant carotid stenosis, and 53% had Fontaine stage II or III claudication. At enrollment, the patients had undergone a total of 57 lower limb revascularizations, and their average age at the time of the procedure was 67 years (66.67 ± 9.84). Of these, 7 were bypass procedures, 49 were angioplasties, and 1 was an endarterectomy. The most common revascularization site was the superficial femoral artery (46%), followed by the iliac artery (25%), the common femoral artery (12%), and the popliteal/tibial arteries (5%). The patients had also undergone a total of 85 carotid revascularizations, with the participants averaging 68 years of age at the time of the intervention (67.55 ± 7.75 years). Of these, 40 were thromboendarterectomies, 40 were percutaneous transluminal angioplasties (PTA), 2 were bypasses, and 3 were combined procedures.

We present the clinical characteristics of the entire cohort in Table 1. The study population demonstrated a high prevalence of overweight, with a mean BMI of 28.4 ± 4.1 kg/m^2^ and a mean waist circumference of 101.22 ± 10.19 cm. The renal function, assessed through eGFR, creatinine, microalbuminuria, and ACR, was within normal limits. The ABI, PWV, and augmentation index were 0.88 ± 0.15, 15.2 ± 7.0 m/s, and 23.17 ± 7.06%, respectively. We found mild increases in fibrinogen (345.07 ± 76.74 mg/dL) and the erythrocyte sedimentation rate (ESR) (25.84 ± 17.15 mm/h). However, the vitamin D levels were low at 16.32 ± 12.22 ng/mL. The mean HOMA-IR was 5.08 ± 8.45, and the mean HOMA-β was 115.49 ± 103.73.

### 3.2. Baseline Characteristics of Group 1 and Group 2

In Table 1, we also present the clinical characteristics of the two groups, according to the median hsCRP levels.

The body composition metrics differed significantly between the groups. Group 2 showed a higher body mass index (29.0 ± 4.3 vs. 27.9 ± 3.7 kg/m^2^; *p* = 0.01), a greater waist circumference (103.32 ± 10.08 vs. 99.19 ± 9.91 cm; *p* < 0.01), and an elevated waist–hip ratio (0.97 ± 0.06 vs. 0.96 ± 0.07; *p* = 0.03) compared to Group 1. However, the cardiovascular parameters remained comparable between the groups, with no significant differences in blood pressure, pulse pressure, or cardiac structural measures, including the interventricular septal thickness, left ventricular posterior wall thickness, ejection fraction, heart rate, and ventricular mass index. The duration of the PR, QRS, and QTc intervals was longer in Group 2, but without reaching statistical significance.

The ABI was higher in patients from Group 2 compared to those from Group 1 (0.90 ± 0.15 vs. 0.86 ± 0.15; *p* = 0.06).

The patients in Group 2 also had higher levels of fibrinogen (361.77 ± 79.84 mg/dL vs. 328.18 ± 69.73 mg/dL; *p* < 0.01) and a higher ESR (29.92 ± 19.39 mm/h vs. 21.71 ± 13.38 mm/h; *p* < 0.01) compared to those in Group 1.

The renal function was worse in Group 2, although not statistically significant, compared to Group 1 (eGFR: 77.07 ± 28.61 mL/min vs. 82.28 ± 26.06 mL/min, *p* = 0.08; serum creatinine: 1.13 ± 0.66 mg/dL vs. 1.02 ± 0.41 mg/dL, *p* = 0.06; ACR: 19.59 ± 47.14 vs. 16.31 ± 61.21, *p* = 0.10).

The lipid profiles were similar between the two groups.

The patients in Group 2, compared to Group 1, had higher levels of glycated hemoglobin (7.12 ± 1.29 vs. 6.78 ± 1.12; *p* = 0.05), HOMA-IR (6.1 ± 10.6 vs. 4.0 ± 5.4; *p* = 0.06), and HOMA-β (132.25 ± 118.85 vs. 99.13 ± 83.61; *p* < 0.01). No differences in vitamin D levels were observed between the two groups.

### 3.3. Baseline Comorbidities and Prior Treatments of the Overall Population

Table 2 presents the baseline comorbidities observed at enrollment, shown both for the entire study population and stratified by median hsCRP levels. Hypertension was the most prevalent comorbidity, affecting 96% of the population, with a mean duration of 11.10 ± 7.70 years, and 84% of the individuals received antihypertensive treatment. Dyslipidemia was present in 95% of the population, with 81% taking lipid-lowering medications. Diabetes affected 47% of the patients, with a mean disease duration of 13.09 ± 10.09 years.

Among the study cohort of 346 participants, 66 individuals (19%) reported a prior cancer diagnosis with an anticipated survival greater than 6 months.

Ischemic heart disease was documented in 113 patients (33%), with a mean age at diagnosis of 62.54 ± 9.66 years. Within this group, 98 participants underwent revascularization procedures: 38 received coronary artery bypass grafting (CABG), 48 underwent a percutaneous coronary intervention (PCI), and 10 had a combined approach involving both surgical and endovascular revascularization techniques.

Cerebral ischemia was clinically diagnosed and confirmed by magnetic resonance imaging in 68 patients (20%), with a mean age at diagnosis of 67.02 ± 10.44 years. Of these, 35 experienced cerebral ictus, 16 had transient ischemic attacks, and 17 exhibited leukoencephalopathy.

At the time of recruitment, carotid revascularization was performed in 85 subjects (25%), with a mean age of 67.55 ± 7.75 years. Of these, nine patients underwent two revascularization procedures. An endarterectomy (TEA) was performed in 40 patients (47%), a percutaneous transluminal angioplasty (PTA) was performed in 40 (47%), a combination of TEA and PTA was performed in 3 (3.5%), and a bypass was performed in 2 (2.4%).

Among the cohort, 57 patients (16%) underwent lower extremity revascularization procedures before recruitment, with a mean age of 66.67 ± 9.84 years. A PTA was the predominant procedure (86%).

A total of 128 patients underwent at least one peripheral arterial revascularization procedure (carotid and/or lower extremity revascularization), with a mean age of 66.98 ± 8.71 years.

The medical treatments prior to enrollment and prescribed at the beginning of the follow-up are summarized in Table 3. A more detailed analysis revealed a significantly higher frequency of prescriptions for antihypertensives, antiplatelet agents, anticoag-ulants, lipid-lowering agents, and antidiabetics after anrollement.

### 3.4. Baseline Comorbidities and Prior Treatments in Group 1 and Group 2

Comorbidities such as hypertension, diabetes, and dyslipidemia were equally distributed between the two groups. Group 2 exhibited a trend of a longer duration of diabetes (14.31 ± 10.91 years vs. 11.77 ± 9.05 years; *p* = 0.15) and a longer duration of hypertension (11.99 ± 7.59 years vs. 10.15 ± 7.73 years; *p* = 0.07) compared to Group 1.

The distribution of cancer patients was similar in both groups. Likewise, no statistically significant differences were observed between the groups in terms of the prevalence of ischemic heart disease, the age at diagnosis, or the rate of myocardial revascularization. Furthermore, the prevalence of cerebral ischemia showed no significant difference between Group 2 and Group 1.

No statistically significant differences in carotid revascularization were observed between the two groups. However, it is noteworthy that a TEA was performed more frequently in Group 2 (26 patients, 57%) compared to Group 1 (14 patients, 36%).

There were no statistically significant differences in lower extremity revascularization procedures prior to enrollment between the two groups. However, a higher percentage of patients in Group 2 underwent bypass procedures (20% in Group 2 vs. 6.3% in Group 1).

Additionally, non significant differences in age or the distribution of peripheral arterial revascularization procedures (carotid and/or lower extremity revascularization) were observed between the two groups.

The medical treatments were similar in both groups.

### 3.5. Outcomes for the Overall Population

After a follow-up of 102.70 ± 44.13 months, we assessed the occurrence of major cardiovascular events, arterial revascularization interventions, and fatal outcomes, as detailed in Table 4.

During the follow-up period, the incidence of ischemic heart disease was 12% (n = 41) and the rate of myocardial revascularization was 13% (n = 44). In nine cases, a second attempt at myocardial revascularization was necessary. The mean interval between the start of follow-up and myocardial revascularization was 33.64 ± 34.07 months for the first and 79.78 ± 41.31 months for the subsequent revascularization.

Cerebral ischemia occurred in 26 patients (7.5%) at a mean follow-up of 48.27 ± 32.79 months.

The incidence of carotid revascularization was 8.1% (n = 28), with a mean follow-up time of 37.33 ± 52.81 months. Two patients required a repeat procedure. Lower extremity revascularization was performed in 29 patients (8.4%) after a mean follow-up of 61.59 ± 45.09 months. No subsequent revascularization procedures were performed on the lower extremities.

At least one peripheral arterial revascularization was performed in 52 patients (15%) during the follow-up period with a mean of 48.39 ± 49.97 months.

During the follow-up period, we documented 78 MAPEs that occurred at 51.02 ± 45.21 months and 129 MACEs with a mean occurrence time of 48.04 ± 43.31 months.

In total, 169 deaths were recorded, representing 49% of the study population.

### 3.6. Outcomes for Group 1 and Group 2

When comparing Group 2 with Group 1 during the follow-up period, no significant differences were observed in the overall incidence of peripheral revascularization (carotid and lower limb procedures).

**Figure 1:**
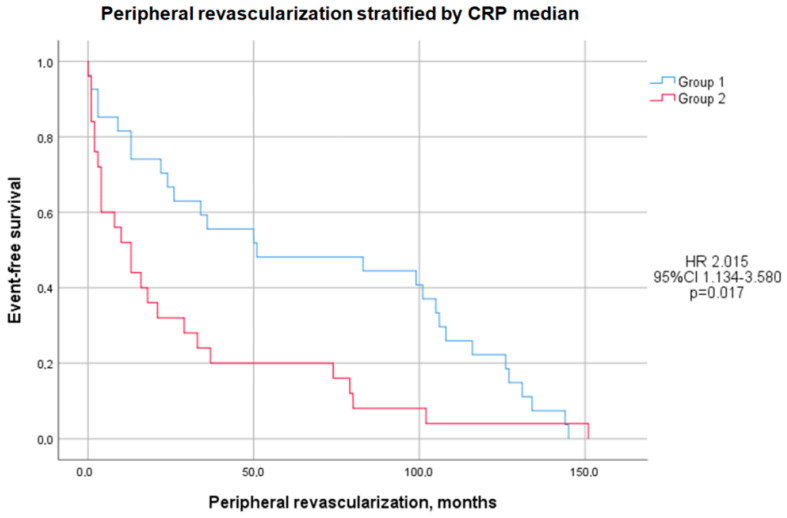
Kaplan-Meier curves comparing peripheral revascularization stratified by median CRP level. The test comparing Group 1 (blue line) and Group 2 (red line) was based on the log-rank test.

Peripheral revascularizations were observed to occur significantly earlier in Group 2 compared to Group 1, with a mean difference of 38.80 ± 65.29 months (28.24 ± 38.87 months vs. 67.04 ± 52.45 months, *p* < 0.01; HR: 2.015, 95% CI: 1.134–3.580, *p* = 0.017), as illustrated in Figure 1. However, this difference lost statistical significance after adjusting for confounding factors, including BMI, the waist-to-hip ratio, fibrinogen, the ESR, HOMA-IR, and HOMA-β. In detail, earlier carotid revascularization was noted in Group 2 (16.80 ± 37.69 months vs. 63.00 ± 59.07 months, *p* = 0.03; HR: 1.827, 95% CI: 0.812–4.112, *p* = 0.145), while lower limb revascularization also tended to occur sooner in Group 2, although this difference did not reach statistical significance (46.36 ± 35.96 months vs. 73.93 ± 48.96 months, *p* = 0.10; HR: 2.467, 95% CI: 1.063–5.723, *p* = 0.035).

Although the total number of MAPEs was similar between the two groups, these events occurred significantly earlier in Group 2 compared to Group 1, with a mean difference of 29.59 ± 60.97 months (36.60 ± 37.35 months vs. 66.19 ± 48.18 months, *p* < 0.01; HR: 1.99, 95% CI: 1.238–3.181, *p* = 0.004). Notably, this difference remained statistically significant even after adjusting for confounding factors such as BMI, the waist-to-hip ratio, fibrinogen, the ESR, HOMA-IR, and HOMA-β (*p* = 0.008).

Additionally, Group 2 demonstrated a strong trend toward an earlier onset of MACEs compared to Group 1, with events occurring 15.58 ± 60.54 months earlier (40.31 ± 38.95 months vs. 55.89 ± 46.33 months, *p* = 0.04; HR: 0.62, 95% CI: 0.983–1.987, *p* = 0.062). After adjusting for BMI, the waist-to-hip ratio, fibrinogen, the ESR, HOMA-IR, and HOMA-β, this trend approached statistical significance (*p* = 0.056).

There were no significant differences between the two groups in the incidence of cerebral ischemia or ischemic heart disease.

**Figure 2:**
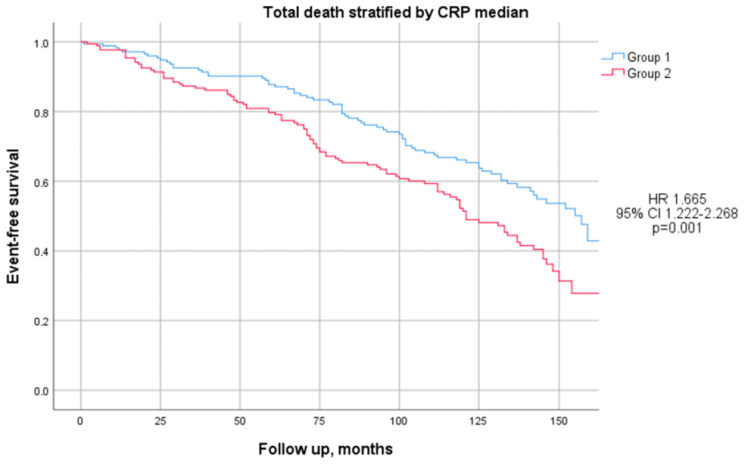
Kaplan-Meier curves comparing total deaths stratified by median CRP level. The test comparing the two groups was based on the log-rank test. The curves showed that Group 2 (red line) had significantly higher mortality with respect to Group 1 (blue line).

However, the mortality was notably higher in Group 2 compared to Group 1 (56% vs. 42%; *p* < 0.01), as illustrated in Figure 2. This difference remained statistically significant after adjusting for confounding factors, including BMI, the waist-to-hip ratio, HOMA-IR, HOMA-β (*p* = 0.04), fibrinogen (*p* = 0.035), and the ESR (*p* = 0.056). Additionally, mortality occurred earlier in Group 2 than in Group 1, although this difference did not reach statistical significance (8.28 ± 43.85 months earlier; 76.58 ± 43.53 months vs. 84.86 ± 5.18 months; *p* = 0.22).

## 4. Discussion

This study provides critical insights into the clinical characteristics and outcomes of patients with advanced peripheral arterial disease (PAD), stratified by high-sensitivity C-reactive protein (hsCRP) levels. By dividing the cohort based on the median hsCRP levels, we identified significant associations between elevated hsCRP and adverse clinical outcomes, including earlier peripheral revascularization and increased mortality.

Inflammation plays a pivotal role in atherosclerosis, and hsCRP, a well-established inflammatory biomarker, has been implicated in the progression of cardiovascular diseases [2,16]. Elevated hsCRP levels were associated with a more pronounced inflammatory profile in our study population, as evidenced by a higher BMI, waist circumference, and waist-to-hip ratio in Group 2 (hsCRP > 0.32 mg/dL). These findings are consistent with studies in the literature linking obesity to systemic inflammation, suggesting that increased adiposity may exacerbate vascular inflammation in PAD [17].

Further supporting the inflammatory hypothesis, Group 2 patients exhibited elevated levels of fibrinogen and a higher erythrocyte sedimentation rate (ESR), markers of systemic inflammation [18,19].

These observations align with emerging evidence highlighting the role of leukotrienes, particularly leukotriene E4 (LTE4) and leukotriene B4 (LTB4), in promoting vascular inflammation and endothelial dysfunction [20]. Leukotrienes, potent inflammatory mediators, have been implicated in the progression of vascular damage by amplifying inflammatory cell recruitment and perpetuating endothelial activation. Unfortunately, leukotrienes were not measured in our study population. Nevertheless, their established role in vascular inflammation represents an important avenue for reflection and a potential area for optimization in future research on PAD. Taken together, our findings underscore the importance of addressing systemic inflammation in patients with PAD, particularly those with elevated hsCRP. The interplay between inflammatory mediators such as hsCRP, leukotrienes, and traditional cardiovascular risk factors, including obesity and hypercoagulability, highlights the need for integrated therapeutic strategies targeting both systemic inflammation and vascular health.

Additionally, the higher HbA1c and HOMA-IR levels in Group 2 reflect poorer glycemic control and greater insulin resistance, both of which are known to contribute to atherogenesis and cardiovascular risk [21].

Although the incidence of traditional cardiovascular comorbidities such as hypertension, diabetes, and dyslipidemia was comparable between the groups, the longer duration of diabetes in Group 2 likely contributed to their heightened inflammatory state. This underscores the importance of cumulative metabolic stress in driving inflammation and cardiovascular risk in PAD [22].

One of the most striking findings of our study was the timing of peripheral revascularization in relation to hsCRP levels. While the overall incidence of revascularization did not significantly differ between the groups, the timing of these interventions was notably earlier in the patients in Group 2, with hsCRP > 0.32 mg/dL, compared to Group 1 patients, with CRP < 0.32 mg/dL. This difference underscores the potential impact of elevated hsCRP on accelerating the progression of atherosclerotic disease [23].

The patients in Group 2 required revascularization significantly sooner than those in Group 1, a pattern observed in both carotid and lower limb procedures, though the degree of statistical significance varied between these vascular territories. This suggests that elevated hsCRP may contribute to the earlier destabilization and progression of atherosclerotic plaques, requiring timely surgical intervention. The earlier need for revascularization in Group 2 highlights the role of systemic inflammation in exacerbating vascular disease [19,24]. Elevated hsCRP levels likely reflect a heightened inflammatory and pro-thrombotic state, which accelerates atherosclerotic progression and plaque vulnerability [23]. This was particularly evident in the earlier carotid revascularizations observed, potentially indicating more rapid disease progression in high-hsCRP individuals. While the trend toward earlier lower limb revascularizations did not reach statistical significance, it suggests regional differences in the impact of systemic inflammation on atherosclerosis [25].

The earlier occurrence of both major adverse cardiovascular events (MACEs) and major adverse peripheral events (MAPEs) in Group 2 highlights the potential role of hsCRP as a prognostic marker for early vascular events. Although the overall incidence of MACEs and MAPEs was similar between groups, their earlier onset in patients with higher hsCRP levels underscores the impact of systemic inflammation on disease progression.

Mortality was significantly higher in Group 2, with a trend toward earlier occurrence, emphasizing the prognostic value of hsCRP in predicting adverse outcomes. This aligns with prior studies that have linked chronic low-grade inflammation, as indicated by elevated hsCRP, to increased all-cause mortality [26,27,28].

Given the inflammatory nature of PAD, anti-inflammatory therapies such as colchicine and IL-1β inhibitors (e.g., canakinumab) are of great interest [5,29]. In contrast to canakinumab, colchicine is an affordable, widely used, and well-established oral anti-inflammatory drug, long recognized for its effectiveness in treating patients with gout, familial Mediterranean fever, and pericarditis [30]. Colchicine has been shown to be a non-targeted anti-inflammatory therapy that can reduce cardiovascular events in patients with established disease [31,32,33,34]. It achieves this through two main mechanisms. Firstly, it inhibits microtubule polymerization, impairing immune cell mobility, adhesion, and activation. Secondly, it disrupts the NLRP3 inflammasome pathway by modulating gene expression and inhibiting caspase-1, thus reducing IL-1β and IL-18 production.

Recent studies have highlighted hsCRP’s role in activating the NLRP3 inflammasome in endothelial cells, linking it to LDL transcytosis and atherosclerosis progression through reactive oxygen species (ROS) regulation and inflammasome activation [35,36]. While colchicine and similar drugs are not yet widely endorsed for broader cardiovascular protection, ongoing studies are investigating their potential in high-risk populations, such as PAD patients, where inflammation is driven by the inflammasome–IL axis. One such study is the LEADER-PAD trial, which is investigating the efficacy of colchicine in reducing the residual cardiovascular risk in patients with peripheral arterial disease (PAD) [37]. This study aims to evaluate whether the anti-inflammatory properties of colchicine can provide additional protection against cardiovascular events in this high-risk population. By targeting the NLRP3 inflammasome and the resulting inflammatory cascade, colchicine may help mitigate the systemic inflammation that contributes to cardiovascular complications in PAD. In patients with peripheral artery disease (PAD), colchicine use has been linked to a reduced risk of major adverse limb events and cardiovascular mortality [38,39].

In conclusion, our findings emphasize the critical role of elevated hsCRP levels in predicting the earlier onset of major adverse cardiovascular events (MACEs) and major adverse peripheral events (MAPEs), including carotid or lower limb revascularization and lower limb amputation, along with higher mortality rates in our study population. These results confirm that hsCRP may be a valuable prognostic marker for assessing cardiovascular and peripheral risks in patients, aiding in the identification of individuals at a higher risk who may benefit from more aggressive management and targeted therapeutic interventions, including lifestyle modifications and anti-inflammatory therapies [40,41]. Incorporating hsCRP measurements into routine clinical practice could enhance risk stratification and management, potentially mitigating the earlier onset of adverse events observed in this high-risk population.

## 5. Study Strengths and Limitations

A key strength of this study is its prolonged follow-up period, which facilitated a detailed evaluation of outcomes such as MACEs, MAPEs, and mortality. Additionally, the study adjusted for several metabolic and inflammatory confounders, strengthening the robustness of our findings regarding the role of hsCRP as an independent predictor of adverse events. This study was designed as a retrospective observational analysis. No a priori power analyses or sample size calculations were performed, as the sample size was determined by the consecutive enrollment of eligible patients who presented to our angiology outpatient clinic during the study period. The consecutive sampling approach was chosen to minimize selection bias while capturing the representative patient population at our institution. Nevertheless, this study has some limitations, including a small sample size and a single-center design, which may restrict the generalizability of its findings. Furthermore, the observational nature of the study and the presence of pre-existing vascular damage and pharmacological treatments make it difficult to establish definitive cause–effect relationships. Furthermore, specific data on the form of lipid-lowering therapy were not characterized or analyzed.

## 6. Conclusions

Our study highlights the significant association between elevated hsCRP levels and the earlier onset of MACEs and MAPEs, alongside an increased mortality, in patients with advanced PAD. These findings underscore hsCRP’s role as a valuable prognostic marker in this population. Elevated hsCRP levels reflect a more adverse metabolic and inflammatory profile, leading to earlier interventions like peripheral revascularization and higher mortality rates. Future research should explore the efficacy of anti-inflammatory therapies targeting hsCRP or its pathways in reducing cardiovascular events and mortality, offering hope for improved management strategies in PAD patients.

## Figures and Tables

**Table 1:** Description of clinical characteristics at baseline.

	All Patients ^1^	Group 1 ^1^	Group 2 ^1^	*p*-Value ^2^
Clinical Parameters				
Men (M)	272 (79%)	135 (78%)	137 (79%)	0.79
Age, yrs	72.11 ± 7.90	71.32 ± 7.65	72.89 ± 8.08	0.07
BMI, Kg/m^2^	28.43 ± 4.06	27.87 ± 3.74	28.99 ± 4.30	0.01
Waist circumference, cm	101.22 ± 10.19	99.19 ± 9.91	103.32 ± 10.08	<0.01
Waist–hip ratio, cm	0.97 ± 0.07	0.96 ± 0.07	0.97 ± 0.06	0.03
Blood Pressure				
Systolic, mmHg	132.08 ± 17.39	132.93 ± 17.58	131.16 ± 17.20	0.37
Diastolic, mmHg	79.61 ± 6.25	80.03 ± 6.63	79.15 ± 5.80	0.21
Mean, mmHg	97.06 ± 8.67	97.61 ± 9.04	96.45 ± 8.22	0.23
PWV, m/s	15.20 ± 6.97	15.22 ± 7.68	15.17 ± 6.17	0.96
Augmentation index, %	23.17 ± 7.06	23.01 ± 6.98	23.34 ± 7.17	0.75
Pulse pressure, mmHg	52.65 ± 15.45	53.24 ± 15.39	52.01 ± 15.54	0.48
Echocardiographic Parameters				
Interventricular septum, mm	11.83 ± 1.33	11.82 ± 1.34	11.85 ± 1.33	0.83
Posterior wall, mm	11.14 ± 1.14	11.14 ± 1.17	11.14 ± 1.12	0.99
Ejection fraction, %	0.58 ± 0.06	0.59 ± 0.05	0.58 ± 0.06	0.35
Ventricular mass index, g/m^2^	77.83 ± 20.29	76.48 ± 18.60	79.20 ± 21.86	0.23
Electrocardiographic Parameters				
Heart rate, bpm	69.82 ± 10.42	70.26 ± 10.50	69.37 ± 10.35	0.43
Sinus rhythm, n (%)	323 (93%)	164 (95%)	159 (92%)	0.28
Right bundle branch block, n (%)	20 (5.8%)	12 (6.9%)	8 (4.6%)	0.36
Left bundle branch block, n (%)	16 (4.6%)	8 (4.6%)	8 (4.6%)	1.00
PR interval, ms	167.99 ± 34.09	166.34 ± 27.89	169.66 ± 39.38	0.39
QRS duration, ms	101.90 ± 21.54	101.26 ± 19.63	102.54 ± 23.32	0.59
QTc, ms	414.00 ± 39.69	411.59 ± 44.01	416.39 ± 34.85	0.28
Blood Chemistry Parameters				
Hemoglobin, g/dL	13.78 ± 1.61	13.86 ± 1.53	13.70 ± 1.70	0.34
Triglycerides, mg/dL	120.60 ± 57.83	120.68 ± 59.43	120.52 ± 56.35	0.95 ^3^
Cholesterol, mg/dL	164.53 ± 39.46	164.42 ± 39.95	164.64 ± 39.09	0.96
HDL cholesterol, mg/dL	48.83 ± 12.50	49.19 ± 12.46	48.47 ± 12.58	0.59
LDL cholesterol, mg/dL	92.75 ± 33.69	92.72 ± 34.01	92.78 ± 33.46	0.99
Fasting glucose, mmol/L	118.87 ± 39.38	119.41 ± 36.49	118.34 ± 42.15	0.80
HbA1c, %	6.95 ± 1.22	6.78 ± 1.12	7.12 ± 1.29	0.05
Serum insulin, mmol/L	19.78 ± 72.29	20.35 ± 98.69	19.19 ± 26.22	0.88
HOMA-IR	5.08 ± 8.45	4.05 ± 5.40	6.12 ± 10.60	0.06 ^3^
HOMA-β	115.49 ± 103.72	99.13 ± 83.61	132.25 ± 118.85	0.01 ^3^
Serum creatinine, mg/dL	1.07 ± 0.55	1.02 ± 0.41	1.13 ± 0.66	0.06
Potassium, mmol/L	4.45 ± 0.44	4.45 ± 0.42	4.46 ± 0.47	0.71
Serum albumin, g/dL	4.54 ± 0.36	4.56 ± 0.35	4.51 ± 0.36	0.22
Fibrinogen, mg/dL	345.07 ± 76.74	328.18 ± 69.73	361.77 ± 79.84	<0.01
ESR, mm	25.84 ± 17.15	21.71 ± 13.38	29.92 ± 19.39	<0.01 ^3^
Vitamin D (25OH), ng/mL	16.32 ± 12.22	16.34 ± 11.87	16.30 ± 12.61	0.98
Urinary Parameters				
Urinary creatinine	97.65 ± 56.16	98.49 ± 49.33	96.82 ± 62.38	0.78
eGFR, mL/min per 1.73 m^2^	79.72 ± 27.43	82.28 ± 26.06	77.07 ± 28.61	0.08
Microalbuminuria, µg/min	118.54 ± 457.89	139.07 ± 619.39	98.38 ± 197.79	0.10 ^3^
ACR	17.87 ± 54.90	16.31 ± 61.21	19.59 ± 47.14	0.10 ^3^

^1^ Median +/− SD or frequency (%); ^2^ Pearson’s chi-squared test or two-sample *t*-test; ^3^ two-sample *t*-test using the logarithm of the non-continuous parameter.

**Table 2:** Description of baseline comorbidities and previous revascularizations.

	All Patients ^1^	Group 1 ^1^	Group 2 ^1^	*p*-Value ^2^
Comorbidity				
Hypertension, n (%)	332 (96%)	166 (96%)	166 (96%)	1.00
Years of hypertension, yrs	11.10 ± 7.70	10.15 ± 7.73	11.99 ± 7.59	0.07
Type 2 diabetes, n (%)	161 (47%)	81 (47%)	80 (46%)	0.91
Years of diabetes, yrs	13.09 ± 10.09	11.77 ± 9.05	14.31 ± 10.90	0.15
Dyslipidemia, n (%)	327 (95%)	165 (95%)	162 (94%)	0.48
Smoking, n (%)	75 (22%)	42 (24%)	33 (19%)	0.24
Cancer, n (%)	66 (19%)	33 (19%)	33 (19%)	1.00
Cardiovascular disease				
Ischemic heart disease, n (%)	113 (33%)	52 (30%)	61 (35%)	0.30
Onset of ischemic heart disease, yrs	62.54 ± 9.66	61.21 ± 8.82	63.84 ± 10.34	0.19
Cerebral ischemia, n (%)	68 (20%)	38 (22%)	30 (17%)	0.28
Onset of cerebral ischemia, yrs	67.02 ± 10.44	64.74 ± 10.86	69.85 ± 9.32	0.05
Type of cerebral ischemia, n (%)				0.49
Ictus	35 (51%)	18 (47%)	17 (57%)	
Leukoencephalopathy	17 (25%)	9 (24%)	8 (27%)	
TIA	16 (24%)	11 (29%)	5 (17%)	
Myocardial revascularization				
Myocardial revascularization, n (%)	98 (28%)	45 (26%)	53 (31%)	0.34
Age at myocardial revascularization, yrs	64.61 ± 8.66	63.79 ± 8.40	65.32 ± 8.90	0.39
Type of revascularization, n (%)				0.36
PCI	48 (50%)	25 (57%)	23 (44%)	
CABG	38 (40%)	14 (32%)	24 (46%)	
CABG + PCI	10 (10%)	5 (11%)	5 (9.6%)	
Limb revascularization				
Lower limb revascularization, n (%)	57 (16%)	32 (18%)	25 (14%)	0.31
Age at lower limb revascularization, yrs	66.67 ± 9.84	65.52 ± 9.01	68.14 ± 10.81	0.32
Type of revascularization, n (%)				0.21
Bypass	7 (12%)	2 (6.3%)	5 (20%)	
PTA	49 (86%)	29 (91%)	20 (80%)	
TEA	1 (1.8%)	1 (3.1%)	0 (0%)	
Site of lower limb revascularization, n(%)				0.33
Common femoral	7 (11%)	2 (6.3%)	5 (17%)	
Iliac	25 (41%)	16 (50%)	9 (31%)	
Popliteal	3 (4.9%)	1 (3.1%)	2 (6.9%)	
Superficial femoral	26 (43%)	13 (41%)	13 (45%)	
Carotid revascularization				
Carotid revascularization, n (%)	85 (25%)	39 (23%)	46 (27%)	0.38
Age at carotid revascularization, yrs	67.55 ± 7.75	68.40 ± 6.48	66.82 ± 8.69	0.35
Type of revascularization, n (%)				0.14
Bypass	2 (2.4%)	2 (5.1%)	0 (0%)	
PTA	40 (47%)	21 (54%)	19 (41%)	
TEA	40 (47%)	14 (36%)	26 (57%)	
TEA + PTA	3 (3.5%)	2 (5.1%)	1 (2.2%)	
Total peripheral revascularizations				
Total peripheral revascularizations, n (%)	128 (37%)	62 (36%)	66 (38%)	0.66
Age at total peripheral revascularizations, yrs	66.98 ± 8.71	66.65 ± 7.96	67.28 ± 9.43	0.68

^1^ Median +/− SD or frequency (%); ^2^ Pearson’s chi-squared test or two-sample *t*-test.

**Table 3:** Description of medical treatments before and after enrollment.

	All Patients ^1^			Before Enrollment			After Enrollment		
Medical Treatments	Before Enrollment	After Enrollment	*p*-Value ^2^	Group 1 ^1^	Group 2 ^1^	*p*-Value ^2^	Group 1 ^1^	Group 2 ^1^	*p*-Value ^2^
Antihypertensive treatment, n (%)	290 (84%)	322 (93%)	<0.01	146 (84%)	144 (83%)	0.77	162 (94%)	160 (92%)	0.67
β-blockers, n (%)	93 (27%)	102 (29%)	0.02	45 (26%)	48 (28%)	0.72	51 (29%)	51 (29%)	1.00
ACE inhibitors, n (%)	132 (38%)	157 (45%)	<0.01	64 (37%)	68 (39%)	0.66	77 (45%)	80 (46%)	0.75
Diuretics, n (%)	149 (43%)	163 (47%)	0.03	71 (41%)	78 (45%)	0.45	72 (42%)	91 (53%)	0.04
ARBs, n (%)	134 (39%)	158 (46%)	<0.01	70 (40%)	64 (37%)	0.51	80 (46%)	78 (45%)	0.83
Calcium channel blockers, n (%)	110 (32%)	120 (35%)	0.07	47 (27%)	63 (36%)	0.06	55 (32%)	65 (38%)	0.26
Nitrates, n (%)	55 (16%)	54 (16%)	0.56	28 (16%)	27 (16%)	0.88	27 (16%)	27 (16%)	1.00
Antiplatelet, n (%)	284 (82%)	317 (92%)	<0.01	142 (82%)	142 (82%)	1.00	160 (92%)	157 (91%)	0.56
Dual antiplatelet therapy, n (%)	255 (74%)	289 (84%)	<0.01	130 (75%)	125 (72%)	0.72	145 (84%)	144 (83%)	0.63
Anticoagulant, n (%)	15 (4.3%)	19 (5.5%)	0.05	7 (4.0%)	8 (4.6%)	0.79	7 (4.0%)	12 (6.9%)	0.24
Lipid-lowering drug, n (%)	280 (81%)	330 (95%)	<0.01	146 (84%)	134 (77%)	0.10	168 (97%)	162 (94%)	0.12
SGLT2i/iDDP4/other, n (%)	0 (0%)	17 (4.9%)	<0.01	0 (0%)	0 (0%)	-	10 (5.8%)	7 (4.0%)	0.46
Subcutaneous insulin, n (%)	13 (3.8%)	58 (17%)	<0.01	5 (2.9%)	8 (4.6%)	0.40	26 (15%)	32 (18%)	0.39
Oral antidiabetic therapy, n (%)	85 (25%)	100 (29%)	0.05	43 (25%)	42 (24%)	0.90	53 (31%)	47 (27%)	0.48
Metformin, n (%)	81 (23%)	90 (26%)	0.04	45 (26%)	36 (21%)	0.25	50 (29%)	40 (23%)	0.22

^1^ Median +/− SD or frequency (%); ^2^ Pearson’s chi-squared test.

**Table 4:** Description of clinical events and revascularizations during follow-up.

	All Patients ^1^	Group 1 ^1^	Group 2 ^1^	*p*-Value ^2^
Follow-up, months	102.70 ± 44.13	108.82 ± 43.56	96.59 ± 43.98	0.01
Death, n (%)	169 (49%)	72 (42%)	97 (56%)	<0.01
Time of death, months	80.11 ± 43.78	84.86 ± 5.18	76.58 ± 43.53	0.22
Cause of death, n (%)				0.63
Cardiovascular events	59 (35%)	28 (39%)	31 (32%)	
Cancer	39 (23%)	15 (21%)	24 (25%)	
Other causes	71 (42%)	29 (40%)	42 (43%)	
Clinical events during follow-up				
Ischemic heart disease, n (%)	41 (12%)	20 (12%)	21 (12%)	0.87
Cerebral ischemia, n (%)	26 (7.5%)	10 (5.8%)	16 (9.2%)	0.22
New cancer diagnosis, n (%)	46 (13%)	20 (12%)	26 (15%)	0.34
Myocardial revascularization during follow-up				
Myocardial revascularization, n (%)	44 (13%)	24 (14%)	20 (12%)	0.52
Time of myocardialrevascularization, months	33.64 ± 34.07	35.50 ± 36.37	31.40 ± 31.88	0.70
Type of revascularization, n (%)				0.28
PCI	34 (76%)	19 (79%)	15 (71%)	
CABG	7 (16%)	2 (8.3%)	5 (24%)	
CABG + PCI	4 (8.9%)	3 (13%)	1 (4.8%)	
Second myocardial revascularization, N (%)	9 (2.6%)	7 (4.0%)	2 (1.2%)	0.09
Time of second myocardial revascularization, months	79.78 ± 41.31	87.29 ± 40.85	53.50 ± 43.13	0.34
Limb revascularization during follow-up				
Lower limb revascularization, n (%)	29 (8.4%)	16 (9.2%)	13 (7.5%)	0.56
Time of lower limb revascularization, months	61.59 ± 45.09	73.94 ± 48.96	46.39 ± 35.96	0.10
Type of revascularization, n (%)				0.18
Bypass	3 (10%)	0 (0%)	3 (23%)	
PTA	23 (79%)	14 (88%)	9 (69%)	
TEA	1 (3.4%)	1 (6.3%)	0 (0%)	
TEA + amputation	2 (6.9%)	1 (6.3%)	1 (7.7%)	
Carotid revascularization during follow-up				
Carotid revascularization, n (%)	28 (8.1%)	13 (7.5%)	15 (8.7%)	0.69
Time of carotid revascularization, months	37.33 ± 52.81	63.00 ± 59.08	16.80 ± 37.69	0.03
Type of revascularization, n (%)				0.47
Bypass	1 (3.6%)	0 (0%)	1 (6.7%)	
PTA	10 (36%)	4 (31%)	6 (40%)	
TEA	16 (57%)	9 (69%)	7 (47%)	
TEA + PTA	1 (3.6%)	0 (0%)	1 (6.7%)	
Second carotid revascularization, n (%)	2 (0.6%)	0 (0%)	2 (1.2%)	0.16
Time of second carotid revascularization, months	83.00 ± 87.68	-	83.00 ± 87.68	-
Peripheral revascularization during follow-up				
Peripheral revascularization, n (%)	52 (15%)	27 (52%)	25 (48%)	0.76
Time of peripheral revascularization, months	48.39 ± 49.97	67.04 ± 52.45	28.24 ± 38.87	<0.01
Major adverse cardiovascular events during follow-up				
MACEs, n (%)	129 (37%)	64 (37%)	65 (38%)	0.91
MACEs, months	48.04 ± 43.31	55.89 ± 46.33	40.31 ± 38.95	0.04
Major adverse peripheral events during follow-up				
MAPEs, n (%)	78 (23%)	38 (22%)	40 (23%)	0.80
MAPEs, months	51.02 ± 45.21	66.19 ± 48.18	36.60 ± 37.35	<0.01

^1^ Median +/− SD or frequency (%); ^2^ Pearson’s chi-squared test or two-sample *t*-test.

## Data Availability

The data supporting the findings are included in the article; further inquiries can be directed to the corresponding author.
